# White Blood Cell, Neutrophil, and Lymphocyte Counts in Individuals in the Evacuation Zone Designated by the Government After the Fukushima Daiichi Nuclear Power Plant accident: The Fukushima Health Management Survey

**DOI:** 10.2188/jea.JE20140092

**Published:** 2015-01-05

**Authors:** Akira Sakai, Tetsuya Ohira, Mitsuaki Hosoya, Akira Ohtsuru, Hiroaki Satoh, Yukihiko Kawasaki, Hitoshi Suzuki, Atsushi Takahashi, Gen Kobashi, Kotaro Ozasa, Seiji Yasumura, Shunichi Yamashita, Kenji Kamiya, Masafumi Abe

**Affiliations:** 1 Dept. of Radiation Life Sciences, Fukushima Medical University School of Medicine, Fukushima, Japan; 2 Dept. of Epidemiology, Fukushima Medical University School of Medicine, Fukushima, Japan; 3 Dept. of Pediatrics, Fukushima Medical University School of Medicine, Fukushima, Japan; 4 Dept. of Radiation Health Management, Fukushima Medical University School of Medicine, Fukushima, Japan; 5 Dept. of Nephrology, Hypertension, Diabetology, and Endocrinology, Fukushima Medical University School of Medicine, Fukushima, Japan; 6 Dept. of Cardiology and Hematology, Fukushima Medical University School of Medicine, Fukushima, Japan; 7 Dept. Gastroenterology and Rheumatology, Fukushima Medical University School of Medicine, Fukushima, Japan; 8 Dept. of Planning and Management, National Institute of Radiological Sciences, Chiba, Japan; 9 Dept. of Epidemiology, Radiation Effects Research Foundation, Hiroshima, Japan; 10 Dept. of Public Health, Fukushima Medical University School of Medicine, Fukushima, Japan; 11 Radiation Medical Science Center for the Fukushima Health Management Survey, Fukushima Medical University School of Medicine, Fukushima, Japan; 12 Japan and Atomic Bomb Disease Institute, Nagasaki University, Nagasaki, Japan; 13 Research Institute for Radiation Biology and Medicine, Hiroshima University, Hiroshima, Japan

**Keywords:** radiation exposure, white blood cells, The Fukushima Health Management Survey

## Abstract

**Background:**

Lymphocytes are susceptible to damage from radiation, and the white blood cell (WBC) count, including counts of neutrophils and lymphocytes, is a useful method of dosimetry. According to the basic survey of the Fukushima Health Management Survey (FHMS), among 13 localities where evacuation was recommended, Iitate and Namie had more individuals with external radiation exposure of more than 5 mSv than the other evacuation areas. We analyzed whether or not WBC, neutrophil, and lymphocyte counts decreased after the disaster.

**Methods:**

The subjects of this study were 45 278 men and women aged 20 to 99 years (18 953 men and 26 325 women; mean age 56 years) in the evacuation zone who participated in the Comprehensive Health Check (CHC) from June 2011 to the end of March 2012.

**Results:**

Significant differences were detected in the mean values of WBC, neutrophil, and lymphocyte counts, and for the proportion of individuals under the minimum standard for WBC and neutrophil counts, among the 13 localities. However, the distribution of individuals at each 200-cell/µL increment in lymphocyte count were similar in these areas, and the WBC, neutrophil, and lymphocyte counts did not decrease in Iitate or Namie specifically.

**Conclusions:**

No marked effects of radiation exposure on the distribution of WBC counts, including neutrophil and lymphocyte counts were detected within one year after the disaster in the evacuation zone.

## INTRODUCTION

The Great East Japan Earthquake that occurred on 11 March 2011 and the resultant Fukushima Daiichi nuclear disaster forced people to evacuate their hometowns, caused them to change their lifestyle to fit a completely new situation, and produced anxiety about radiation. In response to concerns about the effects these factors could have on health, the Comprehensive Health Check (CHC), 1 of 4 detailed surveys of the Fukushima Health Management Survey (FHMS), was implemented to support the prevention of lifestyle-related disease.

Japan experienced atomic bombings in Hiroshima and Nagasaki in 1945. In 1947, the Atomic Bomb Casualty Commission (ABCC) was established to investigate the health impacts on atomic bomb (A-bomb) survivors. Later, a large-scale cohort study of the survivors was started in order to investigate the long-term stochastic effects of radiation. The ABCC had continued to administer follow-up surveys to the present time.^1^^–^^3^ In April 1986, the worst nuclear disaster in human history occurred at the Chernobyl Nuclear Power Plant. The accident released a large quantity of radioactive material into the atmosphere. The USSR Ministry of Health started the Russian National Medical and Dosimetric Registry in June of the same year to register residents exposed to radiation. Unfortunately, however, no epidemiologic studies for evaluating long-term radiation effects on public health were implemented immediately after the accident.^4^

The primary purposes of the FHMS are to monitor the long-term health of residents, promote their future well-being, and determine whether or not long-term low-dose radiation exposure has health effects. The FHMS consists of a basic survey and four detailed surveys, namely: a thyroid ultrasound examination, a comprehensive health check, a mental health and lifestyle survey, and a pregnancy and birth survey.^5^

Lymphocytes are susceptible to damage from radiation, and the evaluation of white blood cell (WBC) count is a useful method of dosimetry. Effective external cumulative doses have been estimated throughout Fukushima Prefecture. As of July 31, 2013, analysis of data from the 435 788 respondents other than radiation workers revealed that, of 1025 respondents with an estimated dose of 5 mSv or greater, 515 had experienced an estimated dose of 10 mSv or greater. Among these, the maximum dose was 25 mSv.^6^ External radiation doses were comparatively high for the Soso regions in Namie and Iitate, located in the evacuation zone designated by the government than the other evacuation areas. Data for respondents other than radiation workers revealed that, of 11 273 Namie residents, 135 had received doses of 5 mSv or greater, among whom 46 had received doses of 10 mSv or greater; among 3170 respondents from Iitate, 800 had received doses of 5 mSv or greater and 70 had received doses of 10 mSv or greater. Although it is impossible to examine the association between external radiation dose and WBC count in individuals at present, these data prompted us analyze whether or not the WBC count, including counts of neutrophils and lymphocytes, decreased in these areas after the disaster in 2011.

## METHODS

Subjects and methods of the CHC have been described by Yasumura previously.^5^ Basically, the CHC performed health examinations for individuals of all ages living in the evacuation zone designated by the government who were officially registered residents at the time of the earthquake. A brief description of the methods follows.

### Subjects

The subjects of this study were residents living in the evacuation zone who were 20 years of age or older. Specifically, the program targeted residents of all areas of Tamura, Minami-Soma, Kawamata, Hirono, Naraha, Tomioka, Kawauchi, Okuma, Futaba, Namie, Katsurao, Iitate, and part of Date, where evacuation was recommended (eFigure 1). A total of 45 278 residents aged 20 to 99 years (18 953 men and 26 325 women; mean age 56 years) in the communities participated in the CHC from June 2011 to the end of March 2012. We excluded residents without data on WBC count (*n* = 28) and those with a past history of or who were being treated for hematologic disease or undergoing dialysis due to renal impairment (*n* = 730). The remaining data on 18 748 men and 25 772 of women were used for the analyses. Informed consent was obtained from the community representatives to conduct an epidemiological study based on guidelines of the Council for International Organizations of Medical Science.^7^ This study was approved by the Ethics Committee of the Fukushima Medical University School of Medicine (approval number 1916).

### Health examinations

The WBC, neutrophil, and lymphocyte counts were added to the Special Health Checkup, which was performed among adults aged 40 years or older in the Fukushima prefecture in 2011, as part of the Municipal National Health Insurance system: In order to analyze the influence of smoking on WBC count, individuals aged 20 years or older were evaluated according to items in the Specific Health Examination, based on the Act on Assurance of Medical Care for Elderly People (Act No. 80, 1982). These items were reported by Yasumura previously^5^ and are listed in eTable 1. Additional items for assessment were serum creatinine (Cr), estimated glomerular filtration rate (eGFR), uric acid (UA), urine testing for occult blood, and peripheral blood count, which included red blood cell (RBC) count, hematocrit (Ht), hemoglobin (Hb), platelet count, and WBC count with subpopulations of white cells. The standard values for WBC count and the ratios of neutrophils or lymphocytes are as follows: WBC, 4.0–9.0 × 10^3^/µL; percentage of neutrophils, 40.0%–70.0%; and percentage of lymphocytes, 20.0%–55.0%. People who reported smoking most days or every day during the previous one month and people who reported having smoked at least 100 cigarettes in total or having smoked for at least 6 months were considered smokers. Information on internal use of medicine was unavailable.

### Statistical analysis

Differences among the localities in mean values for WBC, neutrophil, and lymphocyte counts and prevalence of leukopenia, neutropenia, and lymphopenia were calculated using analysis of covariance or logistic regression models adjusted for age, sex, and smoking status. SAS version 9.3 (SAS Institute, Cary, NC, USA) was used for analyses. All probability values for statistical tests were 2-tailed, and *P* values of less than 0.05 were regarded as statistically significant.

## RESULTS AND DISCUSSION

Upon implementation of the CHC, we expected that there would be no respondents at risk of health impairment due to high-dose radiation exposure according to the results of the estimation of the external dose in the FHMS. However, among 13 localities where evacuation was recommended, Iitate and Namie had more respondents with more than 5 mSv of external radiation exposure than other parts of the evacuation area. The amount of internal radiation exposure in these areas is unknown. To assess the impact of different levels of radiation exposure among the localities of the evacuation area, we analyzed WBC count, including counts of neutrophils and lymphocytes.

**Figure 1.**
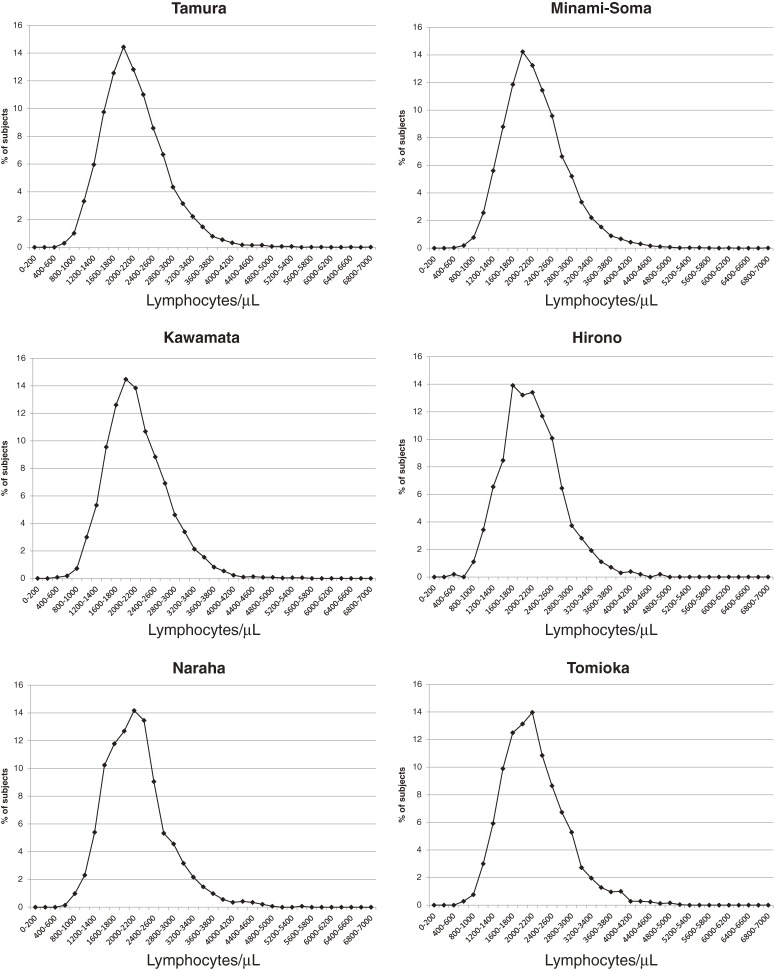

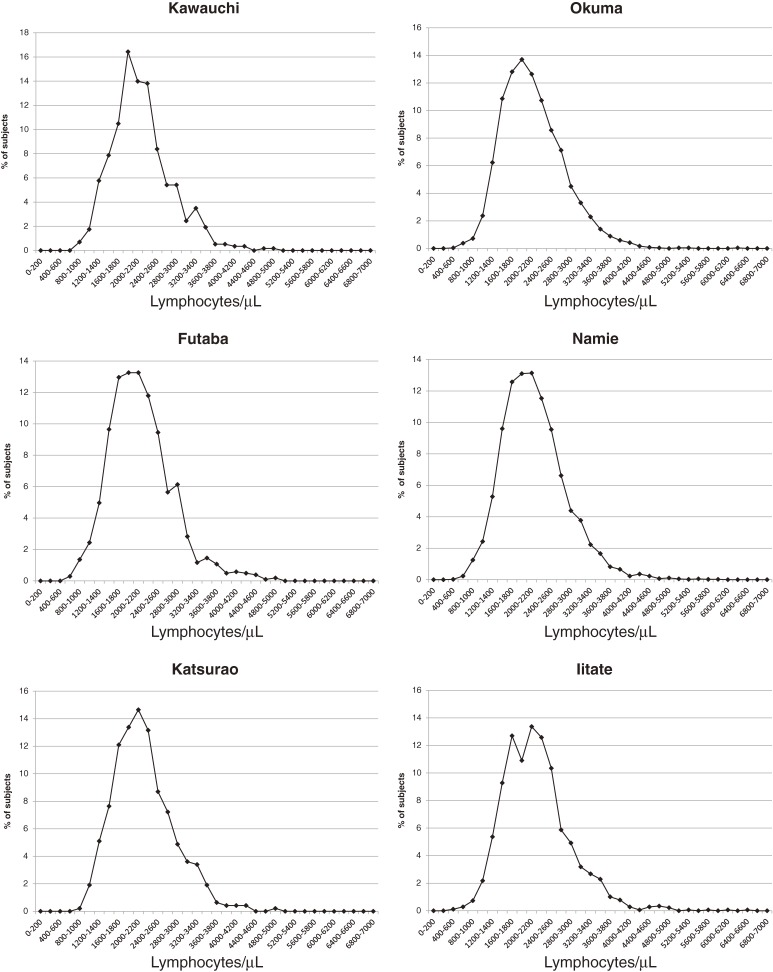

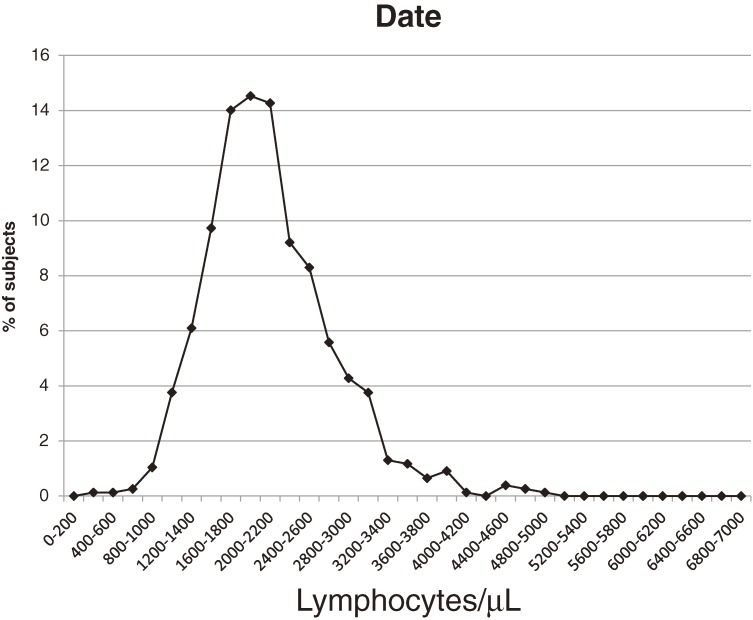
The proportion of individuals for every 200/µL increment of WBC count in 13 localities of the evacuation zone.

In general, after radiation exposure of 250 mSv or higher, lymphocyte counts dip earlier than neutrophil and WBC counts, and neutrophil counts normally increase among smokers. First, we analyzed the number of individuals, stratified by smoking status, gender, and age (<60 or ≥60) who were under the minimum standard counts for WBCs, neutrophils, and lymphocytes in 13 localities. The means of WBC, neutrophil, and lymphocyte counts and the ratios for individuals under the minimal standard count in the 13 localities are shown in Table 1. The proportions of individuals at each 200-cell/µL increment of lymphocytes counts are shown in Figure 1. Significant differences were detected in the mean values of WBC, neutrophil, and lymphocyte counts, and the ratios for individuals under the minimal standard of WBC and neutrophil counts, in the total sample as well as among non-smokers, women, and individuals aged 60 years or older. However, the mean values of WBC, neutrophil, and lymphocyte counts of each locality were within ±5% of the total mean values among the entire sample (data not shown). Moreover, no significant difference was detected in the ratios of individuals under the minimal standard of lymphocyte count in all areas. Further, the distribution of individuals for every 200-cell/µL increment in lymphocyte count were similar in the 13 localities (Figure 1 and eFigure 2), and WBC counts, including neutrophil and lymphocyte counts, were not decreased in Iitate or Namie specifically (Table 1).

**Table 1.** Mean parameter values in 13 localities of the evacuation zone

		Tamura	Minami-Soma	Kawamata	Hirono	Naraha	Tomioka	Kawauchi	Okuma	Futaba	Namie	Katsurao	Iitate	Date	*P for difference*^a^
Total															
*n*		8680	15 646	3894	992	1426	2498	572	2358	1026	4398	471	1788	771	

WBC	Mean (/µL)	5865.28	5986.96	6079.51	5851.11	6019.07	6108.53	6202.62	6010.86	6117.25	5995.41	6076.65	6088.65	6022.18	<0.001
	<4000/µL (%)^b^	9.75	8.22	6.86	9.88	7.71	8.41	5.59	7.97	8.19	8.03	6.58	7.89	8.56	<0.001

Neutrophil	Mean (/µL)	3228.33	3296.10	3411.98	3230.31	3340.14	3445.06	3494.40	3363.07	3438.17	3321.82	3355.52	3351.04	3406.32	<0.001
	<1600/µL (%)^b^	3.72	3.14	2.16	4.03	3.09	3.16	2.27	2.93	3.22	2.77	1.91	3.47	2.98	0.00

Lymphocyte	Mean (/µL)	2124.02	2166.81	2136.40	2110.91	2158.13	2140.07	2173.11	2127.29	2150.44	2158.06	2202.14	2195.81	2083.63	<0.001
	<800/µL (%)^b^	0.29	0.22	0.26	0.20	0.14	0.28	0.00	0.42	0.29	0.25	0.00	0.28	0.52	0.46

Smokers															
*n*		1662	2930	712	152	275	563	106	550	200	928	98	434	132	

WBC	Mean (/µL)	6652.00	6842.00	6702.00	6494.00	6912.00	6975.00	7207.00	6982.00	7097.00	6823.00	7023.00	6879.00	6756.00	<0.001
	<4000/µL (%)^b^	4.15	2.97	3.79	5.26	2.91	3.73	1.89	2.36	2.50	4.09	2.04	2.30	0.76	0.18

Neutrophil	Mean (/µL)	3694.00	3814.00	3765.00	3532.00	3822.00	3950.00	4072.00	3957.00	4076.00	3786.00	3854.00	3857.00	3745.00	<0.001
	<1600/µL (%)^b^	1.32	1.57	1.40	3.29	1.82	1.95	1.89	0.73	0.00	2.26	1.02	1.61	0.76	0.30

Lymphocyte	Mean (/µL)	2338.00	2390.00	2305.00	2351.00	2451.00	2388.00	2489.00	2383.00	2385.00	2412.00	2537.00	2385.00	2356.00	<0.001
	<800/µL (%)^b^	0.06	0.07	0.14	0.00	0.00	0.00	0.00	0.18	0.00	0.00	0.00	0.00	0.76	0.38

Non-smokers															
*n*		7018	12 716	3182	840	1151	1935	466	1808	826	3470	373	1354	639	

WBC	Mean (/µL)	5679.00	5790.00	5940.00	5735.00	5806.00	5856.00	5974.00	5715.00	5880.00	5774.00	5828.00	5835.00	5871.00	<0.001
	<4000/µL (%)^b^	11.07	9.43	7.54	10.71	8.86	9.77	6.44	9.68	9.56	9.08	7.77	9.68	10.17	<0.001

Neutrophil	Mean (/µL)	3118.00	3177.00	3333.00	3176.00	3225.00	3298.00	3363.00	3182.00	3284.00	3198.00	3225.00	3189.00	3336.00	<0.001
	<1600/µL (%)^b^	4.29	3.51	2.33	4.17	3.39	3.51	2.36	3.60	4.00	2.91	2.14	4.06	3.44	<0.001

Lymphocyte	Mean (/µL)	2073.00	2115.00	2099.00	2067.00	2088.00	2068.00	2101.00	2050.00	2094.00	2090.00	2114.00	2135.00	2027.00	<0.001
	<800/µL (%)^b^	0.34	0.25	0.28	0.24	0.17	0.36	0.00	0.50	0.36	0.32	0.00	0.52	0.47	0.58

Men															
*n*		3619	6542	1673	388	556	1064	248	945	437	1898	212	822	344	

WBC	Mean (/µL)	6241.00	6331.00	6275.00	6268.00	6369.00	6424.00	6697.00	6422.00	6514.00	6343.00	6292.00	6391.00	6308.00	<0.001
	<4000/µL (%)^b^	5.06	4.89	5.26	5.67	5.94	5.45	3.23	5.08	5.26	5.06	4.72	5.23	5.23	0.97

Neutrophil	Mean (/µL)	3423.00	3466.00	3496.00	3465.00	3487.00	3588.00	3782.00	3566.00	3650.00	3480.00	3434.00	3493.00	3554.00	<0.001
	<1600/µL (%)^b^	1.82	1.96	1.73	2.06	2.34	1.69	1.21	1.69	1.14	2.11	1.89	2.43	2.62	0.89

Lymphocyte	Mean (/µL)	2241.00	2273.00	2190.00	2217.00	2284.00	2247.00	2287.00	2260.00	2255.00	2279.00	2284.00	2291.00	2145.00	0.00
	<800/µL (%)^b^	0.19	0.12	0.24	0.26	0.00	0.28	0.00	0.42	0.00	0.26	0.00	0.24	0.29	0.64

Women															
*n*		5061	9104	2221	604	870	1434	324	1413	589	2500	259	966	427	

WBC	Mean (/µL)	5596.00	5740.00	5933.00	5584.00	5796.00	5874.00	5824.00	5736.00	5823.00	5732.00	5901.00	5831.00	5792.00	<0.001
	<4000/µL (%)^b^	13.10	10.61	8.06	12.58	8.85	10.60	7.41	9.91	10.36	10.28	8.11	10.14	11.24	<0.001

Neutrophil	Mean (/µL)	3089.00	3174.00	3349.00	3080.00	3246.00	3339.00	3274.00	3227.00	3281.00	3202.00	3291.00	3230.00	3287.00	<0.001
	<1600/µL (%)^b^	5.08	4.00	2.48	5.30	3.56	4.25	3.09	3.75	4.75	3.28	1.93	4.35	3.28	<0.001

Lymphocyte	Mean (/µL)	2040.00	2091.00	2096.00	2043.00	2077.00	2061.00	2086.00	2039.00	2073.00	2066.00	2135.00	2115.00	2034.00	0.00
	<800/µL (%)^b^	0.36	0.29	0.27	0.17	0.23	0.28	0.00	0.42	0.51	0.24	0.00	0.52	0.70	0.76

<60 years															
*n*		4983	8020	2007	475	752	1425	247	1447	560	2341	232	946	429	

WBC	Mean (/µL)	5880.00	6040.00	6116.00	5974.00	6077.00	6184.00	6221.00	6103.00	6167.00	6075.00	6207.00	6200.00	5953.00	<0.001
	<4000/µL (%)^b^	9.89	8.54	6.73	9.68	7.85	8.70	6.07	8.29	8.57	8.63	6.03	6.87	8.86	0.00

Neutrophil	Mean (/µL)	3286.00	3377.00	3463.00	3311.00	3402.00	3525.00	3468.00	3447.00	3506.00	3393.00	3460.00	3468.00	3371.00	<0.001
	<1600/µL (%)^b^	3.35	3.05	2.49	4.63	3.32	3.09	3.64	2.90	3.04	2.78	1.72	2.75	3.50	0.64

Lymphocyte	Mean (/µL)	2074.00	2125.00	2110.00	2142.00	2149.00	2128.00	2216.00	2125.00	2130.00	2154.00	2220.00	2180.00	2044.00	<0.001
	<800/µL (%)^b^	0.28	0.20	0.20	0.21	0.13	0.07	0.00	0.62	0.00	0.13	0.00	0.21	0.70	0.06

≥60 years															
*n*		3697	7626	1887	517	674	1073	325	911	466	2057	239	842	342	

WBC	Mean (/µL)	5846.00	5931.00	6040.00	5738.00	5954.00	6009.00	6189.00	5864.00	6057.00	5904.00	5950.00	5963.00	6108.00	<0.001
	<4000/µL (%)^b^	9.55	7.88	7.00	10.06	7.57	8.01	5.23	7.46	7.73	7.34	7.11	9.03	8.19	0.04

Neutrophil	Mean (/µL)	3151.00	3211.00	3358.00	3156.00	3271.00	3339.00	3514.00	3230.00	3356.00	3240.00	3254.00	3220.00	3450.00	<0.001
	<1600/µL (%)^b^	4.22	3.24	1.80	3.48	2.82	3.26	1.23	2.96	3.43	2.77	2.09	4.28	2.34	<0.001

Lymphocyte	Mean (/µL)	2191.00	2211.00	2165.00	2082.00	2169.00	2157.00	2141.00	2131.00	2175.00	2162.00	2185.00	2213.00	2134.00	<0.001
	<800/µL (%)^b^	0.30	0.24	0.32	0.19	0.15	0.56	0.00	0.11	0.64	0.39	0.00	0.59	0.29	0.42

WBC, white blood cell.

^a^Adjusted for age, sex, and smoking status.

^b^The standard values for WBC count and the ratios of neutrophils or lymphocytes are as follows: WBC 4.0–9.0 × 103/µl; percentage of neutrophils 40.0%–70.0%; and percentage of lymphocytes 20.0%–55.0%.

Based on these results, no effects of radiation exposure on the distribution of WBC counts, including neutrophil and lymphocyte counts, were detected within 1 year after the disaster in the evacuation zone. This result was expected based on the estimation of received external doses in the FHMS, since epidemiological studies have revealed no significant effects on health at doses of 100 mSv or less.^8^

## ONLINE ONLY MATERIALS

eFigure 1. The locations of the 13 evacuated localities in the map of Fukushima prefecture.

eFigure 2. Proportion of individuals for every 200-cell/µL increment of WBC count in all 13 localities.

eTable 1. Items included in the comprehensive health check.

## APPENDIX

The Fukushima Health Management Survey Group includes Masafumi Abe, Shunichi Yamashita, Kenji Kamiya, Seiji Yasumura, Mitsuaki Hosoya, Akira Ohtsuru, Akira Sakai, Shinichi Suzuki, Hirooki Yabe, Masaharu Maeda, Shirou Matsui, Keiya Fujimori, Tetsuo Ishikawa, Tetsuya Ohira, Tsuyoshi Watanabe, Shigeatsu Hashimoto, Kenneth Eric Nollet, Shinichi Niwa, and Yoshisada Shibata.
