# Supplementary figures and images for: White Blood Cell, Neutrophil, and Lymphocyte Counts in Individuals in the Evacuation Zone Designated by the Government After the Fukushima Daiichi Nuclear Power Plant accident: The Fukushima Health Management Survey
Source: J Epidemiol. 2015 Jan 5;25(1):80–7. doi: 10.2188/jea.JE20140092 (PMC4275442; doi:10.2188/jea.JE20140092)

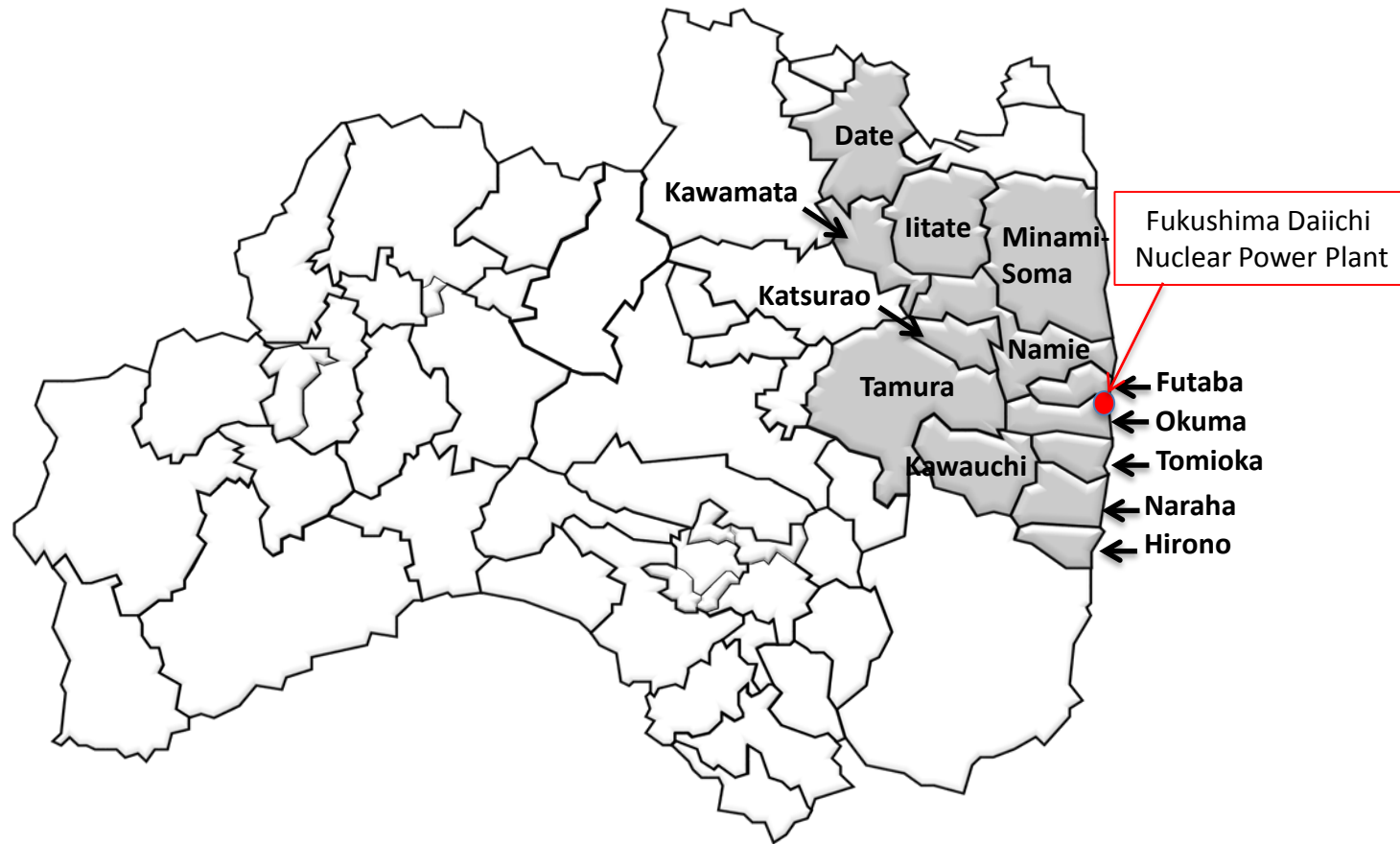

eFigure 1. Locations of the 13 evacuated localities in the map of Fukushima prefecture

Supplement: eFigure 1. [file je-25-080-s001.pdf]

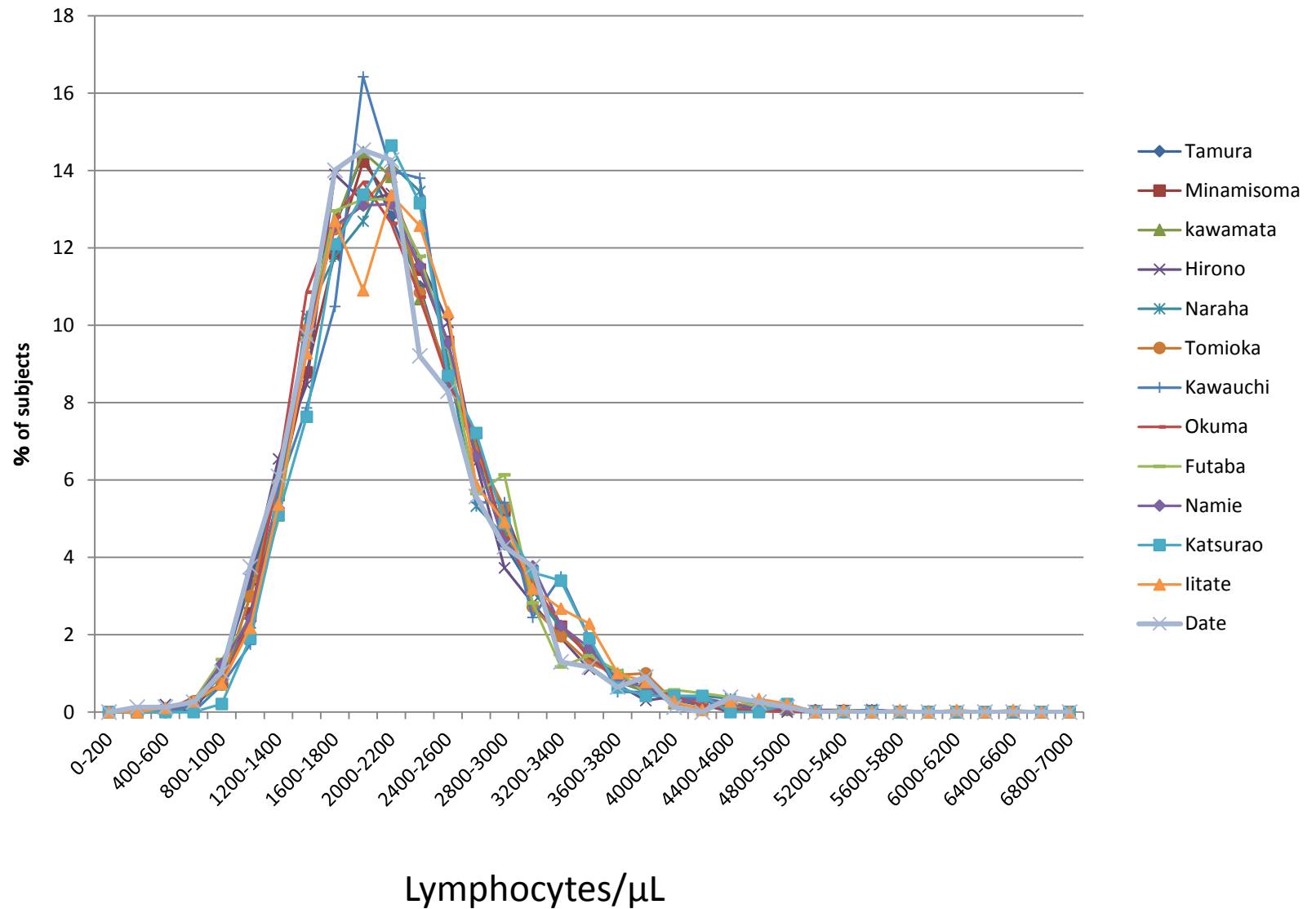

eFigure 2. Proportion of individuals for every 200/μL increment of WBC in all 13 localities

Supplement: eFigure 2. [file je-25-080-s002.pdf]
