# Supplementary material for: White Blood Cell, Neutrophil, and Lymphocyte Counts in Individuals in the Evacuation Zone Designated by the Government After the Fukushima Daiichi Nuclear Power Plant accident: The Fukushima Health Management Survey
Source: J Epidemiol. 2015 Jan 5;25(1):80–7. doi: 10.2188/jea.JE20140092 (PMC4275442; doi:10.2188/jea.JE20140092)
Supplement: eTable 1. [file je-25-080-s003.pdf]

eTable 1. Items included in the comprehensive health check

Age, years	Items
0-6	Height, Weight, Blood cell count (RBC, Hb, Ht, Platelet, WBC, WBC subpopulations)
7-15	Height, Weight, Blood cell count (RBC, Hb, Ht, Platelet, WBC, WBC subpopulations)<br>If requested by patient: Blood chemistry (AST, ALT, γ-GTP, TG, HDL-C, LDL-C, HbA1c, FBG, S-Cr, eGFR, UA)
≥16	Height, Weight, Abdominal circumference/BMI, BP, Blood cell count (RBC, Hb, Ht, Platelet, WBC, WBC subpopulation)<br>Blood chemistry (AST, ALT, γ-GTP, TG, HDL-C, LDL-C, HbA1c, FBS, S-Cr, eGFR, UA)<br>Urinary testing (protein, sugar, blood)

ALT, alanine aminotransferase; AST, aspartate aminotransferase; BMI, body mass index; BP, blood pressure; eGFR, estimated glomerular filtration rate; FBG, fasting blood glucose; γ-GTP, γ-glutamyl transpeptidase; Hb, hemoglobin; HbA1c, hemoglobin A1c; Hct, hematocrit; HDL-C, hight-density lipoprotein-cholesterol; LDL-C, low-density lipoprotein-cholesterol; RBC, red blood cell; S-Cr, serum creatinine; TG, triglyceride; UA, uric acid; WBC, white blood cell.
